# Clinical characteristics of adult T-cell leukemia/lymphoma infiltration in the gastrointestinal tract

**DOI:** 10.1186/s12876-020-01438-1

**Published:** 2020-09-14

**Authors:** Tadashi Miike, Hiroshi Kawakami, Takuro Kameda, Shojiro Yamamoto, Yoshihiro Tahara, Tomonori Hidaka, Yoko Kubuki, Kenji Yorita, Yutaka Akiyama, Yasuji Arimura, Yoshimasa Kubota, Hiroaki Kataoka, Kazuya Shimoda

**Affiliations:** 1 Department of Gastroenterology and Hepatology, Division of Endoscopy and Center for Digestive Disease, University of Miyazaki Hospital, 5200 Kihara, Kiyotake, Miyazaki, 889-1692 Japan; 2 Division of Gastroenterology and Hematology, Department of Internal Medicine, Faculty of Medicine, University of Miyazaki, 5200 Kihara, Kiyotake, Miyazaki, 889-1692 Japan; 3 Division of Gastroenterology and Hepatology, Department of Internal Medicine, Faculty of Medicine, University of Miyazaki, 5200 Kihara, Kiyotake, Miyazaki, 889-1692 Japan; 4 Department of Diagnostic Pathology, Kochi University Hospital, 185-1 Kohasu, Oko-cho, Nankoku, Kochi 783-8505 Japan; 5 Section of Oncopathology and Regenerative Biology, Department of Pathology, Faculty of Medicine, University of Miyazaki, 5200 Kihara, Kiyotake, Miyazaki, 889-1692 Japan; 6 Clinical Research Support Center, University of Miyazaki Hospital, 5200 Kihara, Kiyotake, Miyazaki, 889-1692 Japan

**Keywords:** Adult T-cell leukemia/lymphoma, GI tract infiltration, Irregular blood vessels, Obscure glandular structures, Skin lesion

## Abstract

**Background:**

Adult T-cell leukemia/lymphoma (ATLL) is a peripheral T-cell malignancy caused by human T-cell leukemia virus type 1. The clinical course of ATLL is very heterogeneous, and many organs, including the gastrointestinal (GI) tract, can be involved. However, there are few detailed reports on ATLL infiltration in the GI tract. We investigated the clinical characteristics of ATLL infiltration in the GI tract.

**Methods:**

This retrospective observational single-center study included 40 consecutive ATLL patients who underwent GI endoscopy. The patients’ demographic and clinical characteristics and endoscopic findings were analyzed retrospectively. Patients with ATLL who were diagnosed by histological examination were divided into two groups based on GI tract infiltration.

**Results:**

Multivariate analysis revealed that the absence of skin lesions was significantly associated with GI infiltration (*P* < 0.05). Furthermore, the infiltration group tended to have similar macroscopic lesions in the upper and lower GI tracts, such as diffuse type, tumor-forming type, and giant-fold type.

**Conclusions:**

GI endoscopy may be considered for ATLL patients without skin lesions.

## Background

Adult T-cell leukemia/lymphoma (ATLL) is a peripheral T-cell malignancy caused by human T-cell leukemia virus type 1 (HTLV-1) [1–3]. HTLV-1 infection is usually transmitted vertically, and ATLL develops for several years. The clinical course of ATLL is very heterogeneous, and many organs, including the gastrointestinal (GI) tract, can be involved. B cell malignant lymphoma was recently shown to invade the GI tract and result in clinically detectable findings [4–9]; however, there are few detailed reports on ATLL infiltration into the GI tract. The standard macroscopic classification of ATLL has not yet been established. However, Sakata et al. previously investigated 76 patients of ATLL involvement in the GI tract. They described that the most typical configurations were three types, such as diffuse, tumor-forming, giant fold [10]. This study investigates the characteristics of ATLL infiltration in the GI tract. The current retrospective study was conducted to evaluate whether ATLL infiltration in the GI tract was related to the skin lesion.

## Methods

### Patients and study design

In this retrospective observational study, the demographic and clinical data of 40 consecutive ATLL patients who underwent GI endoscopy between April 1, 2009 and December 31, 2015 at the University of Miyazaki Hospital were analyzed. ATLL was diagnosed according to the Japan Clinical Oncology Group-Lymphoma Study Group criteria (Shinoyama criteria) [11] and classified as four clinical subtypes: acute, lymphoma, chronic, and smoldering. Patients with the acute type are defined as remaining ATLL patients, who usually have leukemic manifestations and tumor lesions. Patients with the lymphoma type are defined as having no lymphocytosis, 1% or more abnormal T lymphocytes, and histologically proven lymphadenopathy with or without extranodal lesions. Patients with the chronic type are defined as having absolute lymphocytosis (≥ 4 × 10^9^/L) with a T lymphocyte count > 3.5 × 10^9^/L; a lactate dehydrogenase value up to twice the normal upper limit; no hypercalcemia; no involvement of the central nervous system, bone, or GI tract; and no ascites or pleural effusion. Further, lymphadenopathy and involvement of the liver, spleen, skin, and lungs may be present, and 5% or more abnormal T lymphocytes are seen in the peripheral blood in most cases. Patients with the smoldering type are defined as having 5% or more abnormal T lymphocytes in the peripheral blood; a normal lymphocyte level (< 4 × 10^9^/L); no hypercalcemia (corrected calcium level < 2.74 mmol/L); a lactate dehydrogenase value up to 1.5 × the normal upper limit; no lymphadenopathy; no involvement of the liver, spleen, central nervous system, bone, or GI tract; and no ascites or pleural effusion. Further, skin and pulmonary lesions may be present, and if there are fewer than 5% abnormal T lymphocytes in the peripheral blood, either histologically proven skin lesions or pulmonary lesions should be present. ATLL diagnoses were made based on anti-HTLV-1 positivity in sera and the presence of malignant mature T-cells.

The study included patients who performed upper GI endoscopy for ATLL. Exclusion criteria included age less than 20 years, a performance status value greater than 4, mental disability, contrast medium allergy, severe heart disease (New York Heart Association class III or IV heart failure), severe pulmonary disease (peripheral oxygen saturation < 90%), actual or possible pregnancy, women wishing to become pregnant, nursing mothers, and refusal to provide informed consent.

Macroscopic GI findings were classified as follows: diffuse type (including multiple ulcers and diffuse erosions), tumor-forming type (including submucosal tumor-like lesion, multiple polypoid lesions, and tumor with ulceration), and giant-fold type, as previously described [10]. Endoscopic examinations were performed using GI endoscopes that emitted white light. If ATLL infiltrated their GI tracts, magnifying endoscopes (GIF-Q240Z, H260Z, PCF-Q260AZI, CF-H260AZI, Olympus, Tokyo, Japan) were used instead of a conventional endoscope. Image-enhanced endoscopy (IEE) findings were assessed by one specialist (TM). We did not perform random biopsies from GI tract. We also performed a small intestinal examination using the whole-body computed tomography (CT), but we did not perform capsule endoscopy or double-balloon endoscopy routinely.

This study was approved by the Research Ethics Committee of the Faculty of Medicine, University of Miyazaki (IRB approval number: O-0003) and performed in accordance with the provisions of the Declaration of Helsinki (as revised in Fortaleza, Brazil, October 2013).

### Statistical analysis

Patients were divided into the infiltration and non-infiltration groups based on whether ATLL infiltrated the GI tract. The definition of ATLL infiltration into the GI tract was both endoscopically and pathologically positive findings. Descriptive statistics such as mean, odds ratios (OR) and 95% confidence intervals (CI) were calculated. Inferential statistical methods such as Chi-squared test and multivariate logistic regression were also applied to assess potential associations between infiltration and non-infiltration, respectively. A *P*-value below 0.05 was considered as the statistical significance level through bivariate analysis and *P* < 0.1 for selecting the variables to introduce into the multivariate regression model. Discrete variables of four clinical subtypes investigated were global assessment of change compared with baseline (4 points ordinal scale: acute = 0, lymphoma = 1, chronic = 2, smoldering = 3) for the order of priority of good prognosis. All analyses were performed using STATA/SE 14.2 (Stata Corp., College Station, TX, USA).

## Results

Patients’ clinical characteristics are summarized in Table 1. All 40 patients who provided consent in this study underwent upper GI endoscopy, while 29 patients underwent both upper and lower GI endoscopy. However, the remaining 11 patinets with poor physical conditions, informed consent could not be obtained for lower GI endoscopy. Positive findings, such as *Candidal* esophagitis, ulcers, erosions, tumor-forming lesions, and diffuse fold lesions, were observed in 22 of the 40 patients who underwent GI endoscopy. Positive findings were observed in 21 of the 40 patients who underwent upper GI endoscopy. Positive findings were observed in 6 of the 29 patients who underwent lower GI endoscopy (Table 2). We observed *Candidal* esophagitis in 5 patients, gastric ulcers in 10 patients, gastric erosions in 1 patient, gastric tumor-forming lesions in 3 patients, diffuse gastric fold lesions in 3 patients, duodenal ulcers in 1 patient, duodenal erosions in 2 patients, colonic ulcers in 2 patients, colonic tumor-forming lesions in 2 patients, and diffuse colonic fold lesions in 2 patients, respectively. Biopsy examinations were subsequently performed for histological diagnosis. Of the 22 patients of the 40 total patients who had upper/lower GI tract endoscopic findings, 12 were diagnosed with ATLL infiltration in the GI tract by histological examination (Table 3). Ten patients were diagnosed with ATLL infiltration in the stomach by histological examination. Two patients had no findings in the stomach. Six patients were diagnosed with ATLL infiltration in the colon by histological examination. Two patients had no findings in the colon. Four patients had none available in the colon. Four of the six patients had both gastric and colonic lesions.

**Table 1:** Characteristics of 40 patients with adult T-cell leukemia/lymphoma

Age, median (range) (years)	67.5 (40–84)
Gender, n (%)/n (%) (male/female)	23 (57.5%) /17 (42.5%)
Digestive symptoms, n (%)/n (%) (+/−)^a^	11 (27.5%) /29 (72.5%)
Clinical subtypes of ATLL, n (%)/n (%)/n (%)/n (%) (acute/lymphoma/chronic/smoldering)	23 (57.5%) /6 (15.0%) /5 (12.5%) /6 (15.0%)
Superficial lymphadenopathy, n (%)/n (%) (+/−)	26 (65.0%) /14 (35.0%)
Hepatosplenomegaly, n (%)/n (%) (+/−)	17 (42.5%) /23 (57.5%)
Skin lesions, n (%)/n (%) (+/−)	26 (65.0%) /14 (35.0%)
Opportunistic infections, n (%)/n (%) (+/−)^b^	15 (37.5%) /25 (62.5%)

^a^Digestive symptoms include nausea, diarrhea, epigastralgia, anorexia, and tarry and bloody stools

^b^Opportunistic infections include herpes zoster, cytomegalovirus infection, pneumonia (mycotic, mycobacterial, pneumocystis), *Candidal* esophagitis, and enteritis

ATLL, Adult T-cell leukemia/lymphoma; +, positive; −, negative

**Table 2:** Proportion of patients with positive findings and adult T-cell leukemia/lymphoma infiltration in the gastrointestinal tract^a^

Examination	Number	Positive findings, % (n/n) 55% (22/40)	ATLL infiltration, % (n/n) 30% (12/40)
Upper GI endoscopy	*n* = 40	53% (21/40)	52% (11/21)
		Esophagus: 5 Stomach: 16 Duodenum: 3	Esophagus: 0 Stomach: 10 Duodenum: 2
Lower GI endoscopy	*n* = 29	21% (6/29)	100% (6/6)
		Small intestine^b^ 5 Large intestine 6	Small intestine^b^ 5 Large intestine 6

^a^Characteristics of upper and lower GI endoscopic findings include duplicative cases

^b^Terminal ileum and cecum

*ATLL* Adult T-cell leukemia/lymphoma, *GI* Gastrointestinal

**Table 3:** Macroscopic findings of 12 patients with adult T-cell leukemia/lymphoma infiltration in the gastrointestinal tract

Case number	Age (years)	Gender	Clinical subtype of ATLL	Macroscopic findings ^a^	
				Stomach	Colon
1	70–80	2	Lymphoma	Diffuse	NA
2	60–70	2	Lymphoma	Tumor-forming	Tumor-forming
3	60–70	1	Acute	Tumor-forming	NA
4	60–70	1	Acute	Diffuse	Diffuse
5	70–80	1	Acute	Diffuse	Diffuse
6	60–70	1	Acute	Tumor-forming	No findings
7	60–70	2	Acute	Diffuse	NA
8	70–80	2	Acute	Giant-fold	Giant-fold
9	70–80	1	Acute	No findings	Giant-fold
10	70–80	2	Acute	Giant-fold	No findings
11	80–90	2	Lymphoma	Diffuse	NA
12	60–70	2	Acute	No findings	Tumor-forming

The identifying information (e.g. age and gender) were anonymized

^a^ Macroscopic GI findings were classified as follows: diffuse type, tumor-forming type, and giant-fold type, as previously described [10]

*ATLL* Adult T-cell leukemia/lymphoma, *NA* not available

**Fig. 1:**
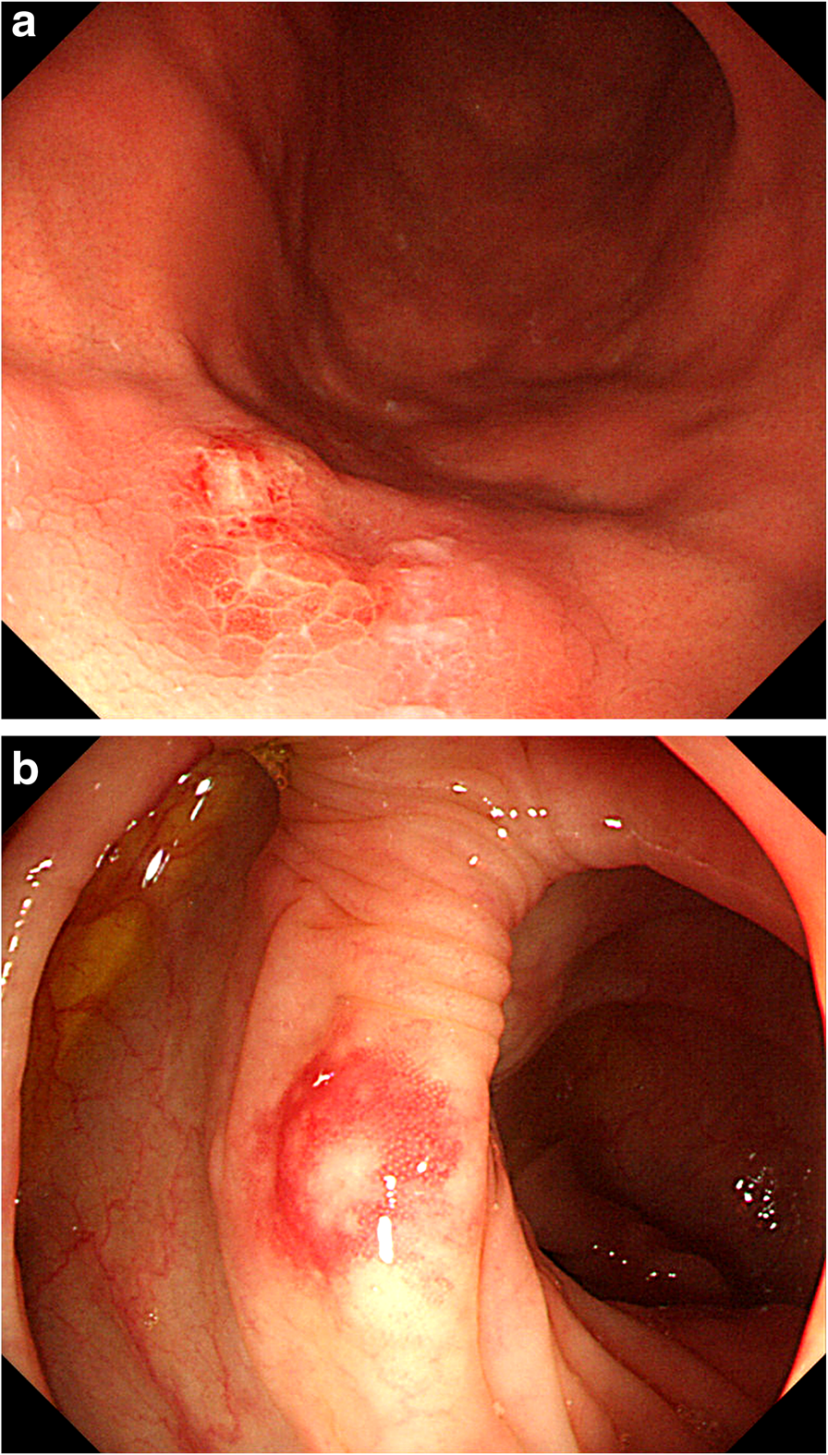
Macroscopic gastrointestinal findings. **a** A 60s-year-old person underwent endoscopy for screening purposes. Upper gastrointestinal (GI) endoscopy shows scattered reddish mucosal protrusions on the greater curvature of the gastric body. **b** Colonoscopy image shows sporadic protrusions

**Fig. 2:**
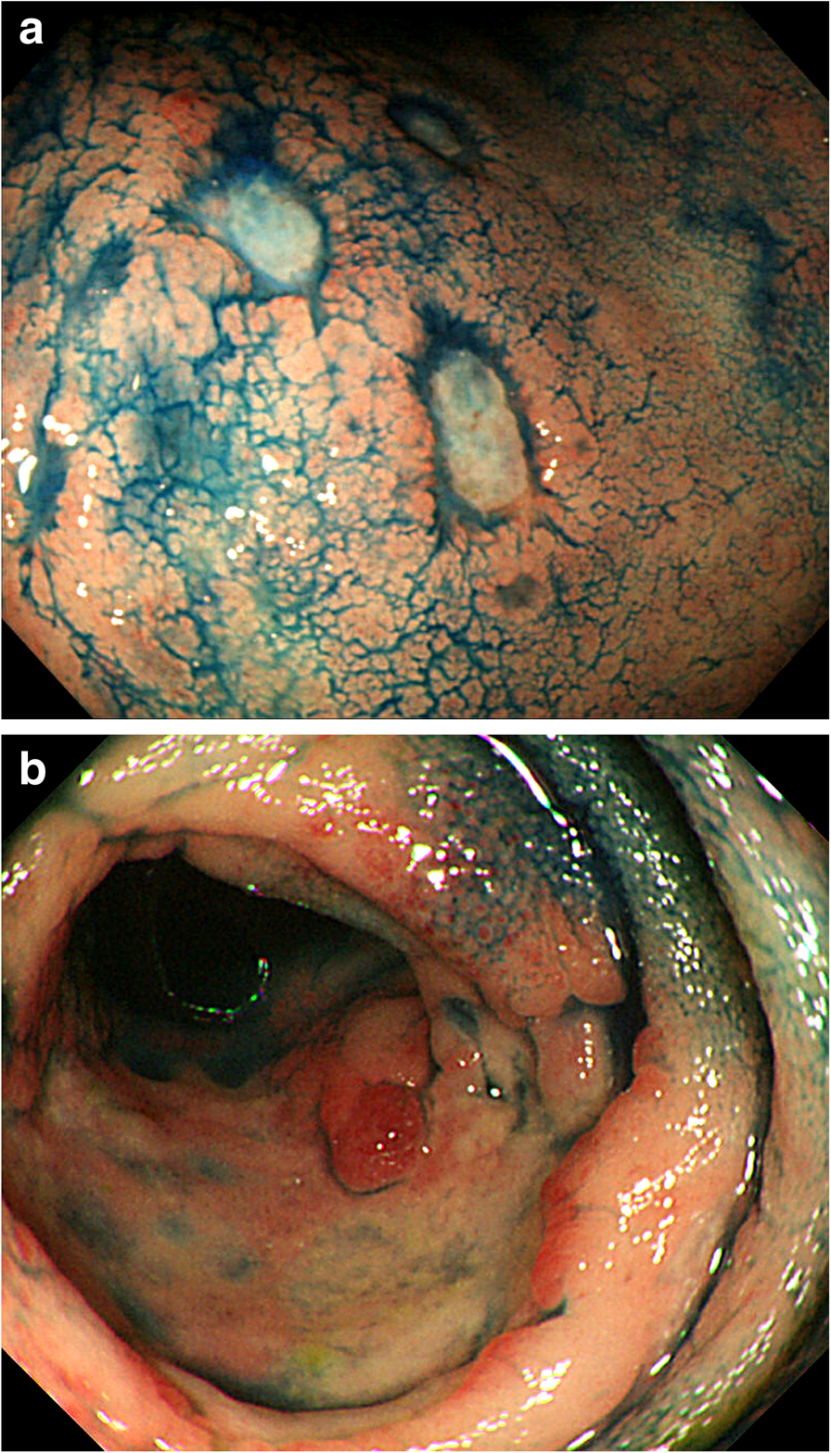
Macroscopic gastrointestinal findings. **a** A 70s-year-old person underwent endoscopy due to a tarry stool. Upper GI endoscopy shows diffusely ulcerative mucosa on the anterior wall of the gastric body. **b** Colonoscopy image shows sporadic ulceration

**Fig. 3:**
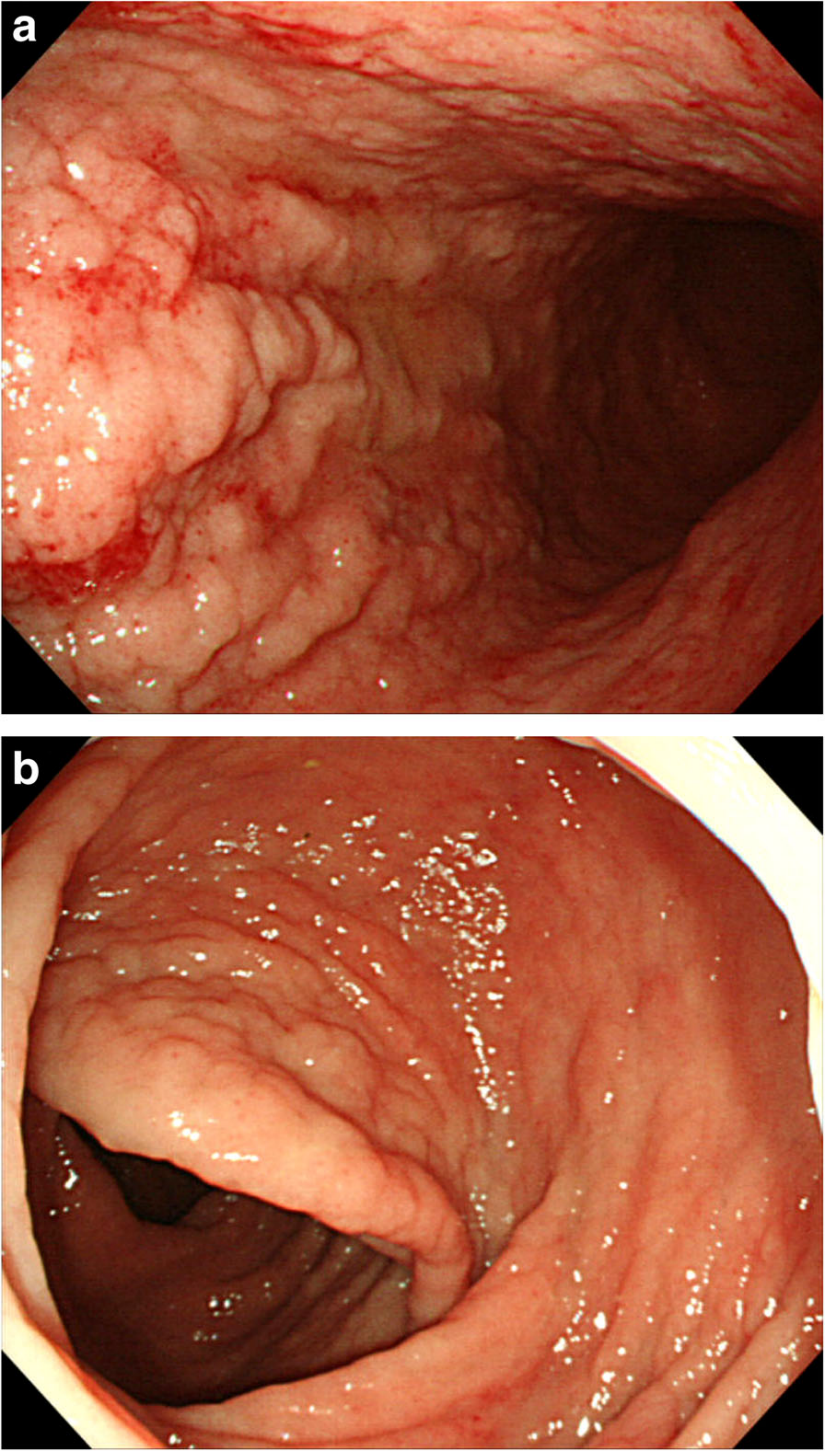
Macroscopic gastrointestinal findings. **a** A 70s-year-old person underwent endoscopy due to diarrhea. Esophagogastroduodenoscopy shows diffuse areas of coarse mucosal granularity on the greater curvature of the gastric body. **b** Colonoscopy image shows sporadic diffuse granularity

**Fig. 4:**
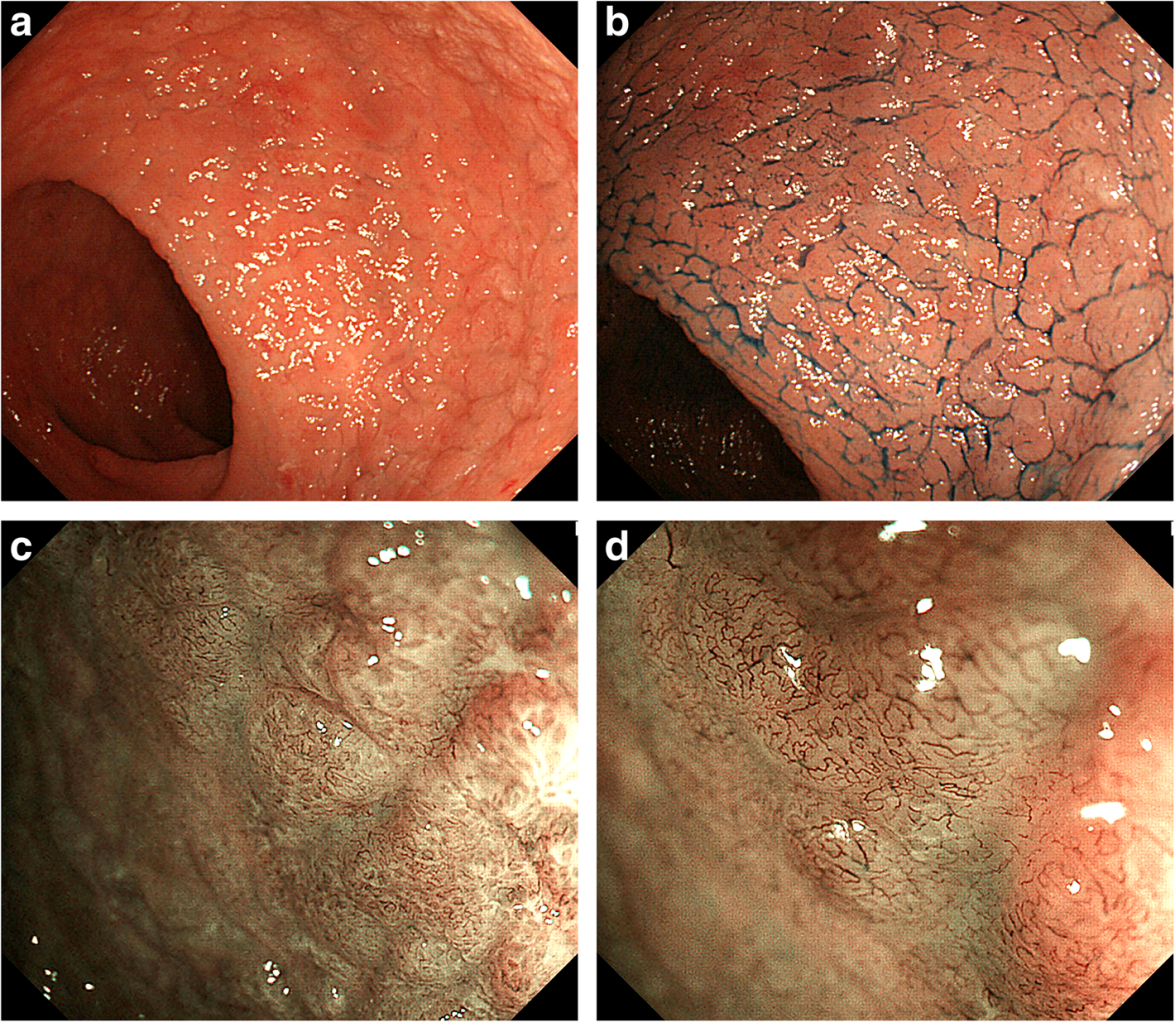
Magnified endoscopic images on Image-enhanced endoscopy. **a** A 70s-year-old person underwent endoscopy due to epigastralgia and diarrhea. Colonoscopy shows diffuse areas of coarse mucosal granularity. **b** Chromoendoscopy with indigo carmine clearly shows coarse mucosal granularity. **c** Magnified endoscopic examination with narrow-band imaging (NBI) reveals obscure glandular structures. **d** Magnified endoscopic examination with NBI reveals irregular bifurcated meandering blood vessels

All 40 patients underwent CT and ^18^F-fluorodeoxyglucose (FDG) positron emission tomography-CT, respectively. One patient revealed an abdominal uptake of FDG in the ileum. Therefore, we performed double-balloon endoscopy and diagnosed diffuse type of ATLL in the small intestine, which was in concordance with abnormal uptake of FDG. Upper GI endoscopy showed findings such as scattered reddish protruding lesions, diffuse ulcerative lesions, and localized/diffuse areas of coarse mucosal granularity (Figs. 1, 2 and 3). Five patients only underwent IEE because of non-overt subjective symptoms and progression of illness at this time. IEE showed cases of obscure glandular structures and irregular bifurcated meandering blood vessels (Fig. 4). Lower GI endoscopy showed similar colonic findings to IEE.

All ATLL patients did not have GI adverse events during chemotherapy. Thus, we could not examine the endoscopic follow-up with a poor prognosis.

Univariate analysis showed no significant difference between the infiltration and non-infiltration groups in terms of clinical features (Table 4). However, digestive symptoms (*P* = 0.045) and acute-type and lymphoma-type ATLL (*P* = 0.053) were more prevalent in the infiltration group, and skin lesions were significantly more prevalent in the non-infiltration group (*P* = 0.009). Multivariate analysis revealed that ATLL infiltration in the GI tract was significantly associated with the absence of skin lesions (*P* = 0.041) (Table 5).

**Table 4:** Comparison of patients with and without adult T-cell leukemia/lymphoma infiltration in the gastrointestinal tract

Characteristics	Infiltration group^a^	Non-infiltration group^b^	*P*-value
	*n* = 12	*n* = 28	
Age, median (range) (years)	69 (61–82)	66.5 (40–84)	0.585^c^
Gender, n (%)/n (%) (male/female)	5 (41.7%) /7 (58.3%)	18 (64.3%) /10 (35.7%)	0.185^d^
Digestive symptoms, n (%)/n (%) (+/−)	6 (50.0%) /6 (50.0%)	5 (17.9%) /23 (82.1%)	0.037^d^
Clinical subtypes of ATLL, n (%)/n (%)/n (%)/n (%) (acute/lymphoma/chronic/smoldering)	9 (75.0%) /3 (25.0%) /0 (0%) /0 (0%)	14 (50.0%) /3 (10.7%) /5 (17.8%) /6 (21.4%)	0.080^d^
Superficial lymphadenopathy, n (%)/n (%) (+/−)	7 (58.3%) /5 (41.7%)	19 (67.9%) /9 (32.1%)	0.563^d^
Hepatosplenomegaly, n (%)/n (%) (+/−)	6 (50.0%) /6 (50.0%)	11 (39.3%) /17 (60.7%)	0.530^d^
Skin lesions, n (%)/n (%) (+/−)	4 (33.3%) /8 (66.7%)	22 (78.6%) /6 (21.4%)	0.006^d^
Opportunistic infections, n (%)/n (%) (+/−)	4 (33.3%) /8 (66.7%)	11 (39.3%) /17 (60.7%)	0.722^d^

^a^Group of ATLL patients with infiltration in the gastrointestinal tract

^b^Group of ATLL patients without infiltration in the gastrointestinal tract

c Wilcoxon (Mann-Whitney) test

^d^ Chi-squared test

*ATLL* Adult T-cell leukemia/lymphoma

**Table 5:** Statistical analysis of patients with and without adult T-cell leukemia/lymphoma infiltration in the gastrointestinal tract

		Univariate analysis			Multivariate analysis	
	Odds ratio	95% CI	*P*-value	Odds ratio	95% CI	*P*-value
Age	0.397	0.099–1.583	0.190	–		
Gender	1.026	0.962–1.095	0.436	–		
Digestive symptoms	4.600	1.038–20.381	0.045	2.443	0.423–14.106	0.318
Clinical subtypes of ATLL^a^	0.375	0.139–1.014	0.053	0.468	0.165–1.329	0.154
Superficial lymphadenopathy	0.663	0.164–2.676	0.564	–		
Hepatosplenomegaly	1.545	0.396–6.035	0.531	–		
Skin lesions	0.136	0.030–0.6035	0.009	0.184	0.036–0.934	0.041
Opportunistic infections	0.773	0.187–3.196	0.722	–		

*ATLL* Adult T-cell leukemia/lymphoma, *CI* Confidence interval

^a^ Four clinical subtypes investigated were global assessment of change compared with baseline (4 points ordinal scale: acute = 0, lymphoma = 1, chronic = 2, smoldering = 3) for the order of priority of good prognosis

## Discussion

ATLL is a systemic disease with an unfavorable prognosis, and any organ can be impaired by ATLL cell infiltration. We observed endoscopic characteristic findings of ATLL infiltration in the GI tract in 12 patients (30%). Previous studies have also reported a prevalence of about 30% [10, 12]. Moreover, in our study, IEE showed that ATLL infiltration in the GI tract is characterized by obscure glandular structures and irregular bifurcated meandering blood vessels.

Our study presents several novel findings. Firstly, we observed a correlation between gastric and colonic lesions. Nakamura et al. previously proposed that, in GI infiltration, the stomach and intestine would present different ATLL lesion types [13]. However, our study shows similar macroscopic findings in the stomach and colon, even though it is generally accepted that the macroscopic forms of ATLL vary throughout the GI tract. Therefore, endoscopy is necessary for both regions to closely evaluate GI tract infiltration. Secondly, we observed obscure glandular structures and irregular bifurcated meandering blood vessels on the magnified endoscopic images of five ATLL patients with GI tract infiltration. These findings seem characteristic of lymphoproliferative diseases. Nonaka et al. [4] found that magnified endoscopic images from the narrow-band imaging of gastric mucosa-associated lymphoid tissue lymphoma present obscure glandular structures and irregular blood vessels with minimal changes in size and caliber, despite lymphoma cell infiltration. These common features between B cell lymphoma and ATLL may be caused by lymphoma cell infiltration in stromal tissue, the concomitant destruction of glandular structures, and neoplastic growth. The displacement of superficial vessels caused by proliferating lymphoid tissue may have resulted in the minimal size and caliber changes and meandering observed. Moreover, the destruction of glandular structures may be caused by the initial diffuse permeation of ATLL cells around the muscularis mucosa, followed by the aforementioned changes in blood and lymphatic vessels.

Furthermore, we found that patients with digestive symptoms, and no skin lesions may have a high risk of GI tract infiltration. Our multivariate analysis showed that the absence of skin lesions significantly correlated with ATLL infiltration in the GI tract. Skin lesions have been observed in about half of all ATLL patients [11] and are more prevalent in patients with chronic-type or smoldering-type ATLL than in those with acute-type or lymphoma-type ATLL. Additionally, aggressive ATLL may be more likely to infiltrate the GI tract [14–18]. We recommended physicians be more attentive to the lack of skin lesions in ATLL patients.

Although this study provides useful data regarding ATLL infiltration in the GI tract, it has several limitations related to its observational, non-randomized, single-center, and retrospective design. Firstly, selection bias for both groups could not be avoided because there were no assessments of real-time diagnosis performance. Secondly, because ATLL has an unfavorable prognosis, it was difficult to perform GI endoscopy multiple times when investigating GI tract infiltration. Thirdly, IEE findings were assessed by one specialist at a single institution. Fourthly, the number of cases was limited. Fifthly, All ATLL patients did not undergo colonoscopy. More cases are needed to clarify the usefulness of IEE in diagnosing ATLL infiltration in the GI tract.

## Conclusions

Physicians may perform GI endoscopy to actively investigate ATLL infiltration in the GI tract and evaluate the endoscopic findings characteristic of lymphoproliferative disease. The absence of skin lesions and the presence of characteristic endoscopic findings may help detect ATLL infiltration in the GI tract. GI endoscopy may be considered for ATLL patients without skin lesions.

## Data Availability

The datasets used and/or analysed during the current study are available from the corresponding author (TM) on reasonable request.

## Abbreviations

ATLL: Adult T-cell leukemia/lymphoma
HTLV-1: Human T-cell leukemia virus type 1
GI: Gastrointestinal
IEE: Image-enhanced endoscopy
CT: Computed tomography
OR: Odds ratio
CI: Confidence interval
FDG 18F: fluorodeoxyglucose
NA: Not available
NBI: Narrow band imaging
